# Whole-Genome Shotgun Sequence of *Pseudomonas viridiflava*, a Bacterium Species Pathogenic to *Arabidopsis thaliana*

**DOI:** 10.1128/genomeA.00116-12

**Published:** 2013-01-24

**Authors:** Francois Lefort, Gautier Calmin, Julien Crovadore, Magne Osteras, Laurent Farinelli

**Affiliations:** a Plants and Pathogens Group, Research Institute Earth Nature and Environment, hepia, University of Applied Sciences of Western Switzerland, Jussy, Switzerland; b Fasteris SA, Ch. du Pont-du-Centenaire CH-Plan-les-Ouates, Switzerland

## Abstract

We report here the first whole-genome shotgun sequence of *Pseudomonas viridiflava* strain UASWS38, a bacterium species pathogenic to the biological model plant *Arabidopsis thaliana* but also usable as a biological control agent and thus of great scientific interest for understanding the genetics of plant-microbe interactions.

## GENOME ANNOUNCEMENT

*Pseudomonas viridiflava* is a pectinolytic bacterium member of the *Pseudomonas syringae* group (1). It is pathogenic to numerous cultivated crops and weeds (2), including *Arabidopsis thaliana*, in which it induces both compatible (disease) and incompatible (resistance) responses (3). For this reason, it has triggered much interest in plant-microbe interaction studies in *A. thaliana* (4, 5).

Pathogenicity genes and mechanisms are becoming increasingly well-known, and 2 paralogous pathogenicity islands (T-PAI and S-PAI), which share many gene homologs, have been described for *P. viridiflava* (6, 7).

*P. viridiflava* was shown to display a high level of genetic variation worldwide, with all isolated *P. viridiflava* strains parting into two distinct and deeply diverged clades, with evidence of frequent recombination but little geographic differentiation (4, 5). These 2 distinct clades cause disease symptoms of differing severities.

This bacterium is an antimycotic producer that is usable in biological control against other plant pathogens (8), and this strain was evaluated as a biological control agent against postharvest disease of pip fruits (9).

The *P. viridiflava* strain UASWS0038 was isolated in our lab from a *Phytophthora* sp.-infected rhododendron leaf. Axenic isolates were submitted to DNA extraction according to a modified DNA extraction micromethod (10). Whole-genome shotgun sequencing of the *P. viridiflava* strain UASWS0038 was then carried out in an IIlumina genome analyzer II, producing 6,317,750 paired-end reads that were 36 bp long and 8,441,093 single reads that were 35 bp long. Assembly was carried out with ABySS 1.3.4 (11). This led to 201 contigs, for a genome length of 5,910,810 bp, and yielded a contig N_50_ of 48,957. This assembly was run in RAST 4.0 (12). Rapid Annotation using Subsystem Technology (RAST) analysis identified 66 RNA genes and 5,340 coding DNA sequences, of which more than half could be allocated a function. It is estimated that there are approximately 46 missing genes. Annotation was carried out upon submission using the Prokaryotic Genomes Automatic Annotation Pipeline Group (PGAAPG).

Regarding nitrogen metabolism, this strain is equipped for nitrate and nitrite ammonification and for ammonium assimilation. An amidase-urea-nitrile hydratase cluster would allow strain UASWS0038 to utilize monocarboxylic acid amide, formamide, urea, and nitriles.

This strain also contains genes conferring resistance to semimetals and metals, such as arsenic, chromium, copper, cobalt, zinc, and cadmium. No plasmid sequence was found, but a complete prophage genome was detected. UASWS0038 also has genes involved in lysozyme inhibition, a multidrug resistance tripartite typical of Gram-negative bacteria, multidrug resistance efflux pumps, a multidrug efflux system, and streptomycin and fluoroquinolone resistance, as well as beta-lactamase synthesis. The strain displays a few secretory protein genes, such as *hrpF*, *hrpS hrpZ*, and *hrpW*, but no complete pathogenicity islands were found that would make this strain usable as a biological agent.

In-depth study of the genome of *P. viridiflava* strain UASWS0038 and comparisons to the genomes of pathogenic strains would help to elucidate the mechanisms of coevolution in natural plant-pathogen interactions.

### Nucleotide sequence accession number.

This whole-genome shotgun project was deposited at GenBank under the accession no. AMQP00000000 (GenBank Assembly ID: GCA_000307715.1; RefSeq Assembly ID: GCF_000307715.1).
